# Inferring the reproduction number using the renewal equation in heterogeneous epidemics

**DOI:** 10.1098/rsif.2021.0429

**Published:** 2022-03-30

**Authors:** William D. Green, Neil M. Ferguson, Anne Cori

**Affiliations:** ^1^ Infectious Disease Epidemiology, Imperial College London, London, UK; ^2^ MRC Centre for Global Infectious Disease Analysis, Imperial College London, London, UK; ^3^ Abdul Latif Jameel Institute for Disease and Emergency Analytics, Imperial College London, London, UK

**Keywords:** heterogeneous, epidemics, renewal equation, generation time, asymptomatic transmission

## Abstract

Real-time estimation of the reproduction number has become the focus of modelling groups around the world as the SARS-CoV-2 pandemic unfolds. One of the most widely adopted means of inference of the reproduction number is via the renewal equation, which uses the incidence of infection and the generation time distribution. In this paper, we derive a multi-type equivalent to the renewal equation to estimate a reproduction number which accounts for heterogeneity in transmissibility including through asymptomatic transmission, symptomatic isolation and vaccination. We demonstrate how use of the renewal equation that misses these heterogeneities can result in biased estimates of the reproduction number. While the bias is small with symptomatic isolation, it can be much larger with asymptomatic transmission or transmission from vaccinated individuals if these groups exhibit substantially different generation time distributions to unvaccinated symptomatic transmitters, whose generation time distribution is often well defined. The bias in estimate becomes larger with greater population size or transmissibility of the poorly characterized group. We apply our methodology to Ebola in West Africa in 2014 and the SARS-CoV-2 in the UK in 2020–2021.

## 1. Introduction

The effective reproduction number, *R*, defined as the average number of secondary infections generated by each primary case, is of fundamental importance in infectious disease epidemiology. When *R* is above 1, infection prevalence is expected to increase, whereas when *R* is below 1, it will decline. As such, interventions for epidemic control generally aim to reduce the *R* to below unity.

Estimation of *R* has taken on particular significance over the past year in light of the global COVID-19 pandemic, which is so far responsible for over 200 million cases, and 4.5 million deaths worldwide [1]. Given the importance of *R* in elucidating the extent of control measures required to suppress the epidemic, real-time estimation of *R* has been the focus of disease modelling groups and government health departments worldwide [2].

The effective reproduction number principally depends on the transmission ability of the pathogen in a totally susceptible population (one with no existing immunity) and the level of immunity in the population. The transmission ability is often represented by the basic reproduction number, *R*_0_, defined as the average number of secondary infections arising from a primary case in a large, totally susceptible population. *R* may be further modified by changes in the number, frequency and closeness of contacts in a population, hygiene practices, seasonal variation, population demographics and pathogen evolution. *R* is generally estimated from trends in infections, cases, hospitalizations or deaths over time [3–6].

There are two distinct reproduction numbers than can be derived from data on infection incidence. The instantaneous reproduction number, henceforth denoted *R*(*t*), represents the average number of individuals someone infected at time *t* would infect if conditions remained unchanged. Conversely, the case reproduction number, *R_c_*(*t*), represents the average number of people an individual infected at time *t* actually infects, which will depend on changes in policy or behaviour over the period of that cohort's infection, and can thus only be estimated in retrospect [7,8]. The work of this paper focusses on the former, which is better suited to track changes in transmission in real time, and which will reduce immediately following the start of a successful intervention [7].

Prompted by the SARS-CoV-2 pandemic, there has been a significant body of recent work considering the optimal approach for estimating *R*, and potential sources of error. A simple and widely used approach to estimate the effective reproduction number uses the renewal equation, which uses as inputs the generation time distribution, *ω*(*τ*) (the distribution of times, *τ*, between infection in a case and infection of their infector) and the time-series of infection incidence [7]. This method only considers the average reproduction number and a single generation time distribution across all infected individuals.

However, heterogeneity in transmission may result from biological and behavioural differences between individuals. An example of a biological difference between individuals is symptomatic and asymptomatic infection. Similarly, a subset of symptomatic individuals may change their behaviour to limit their social contacts (self-isolation), as is currently mandated in UK law for both confirmed SARS-CoV-2 cases and their immediate household. Heterogeneity may also arise owing to the deployment of novel pharmaceuticals (e.g. antiretroviral therapy) or through vaccination priming the immune response in a subset of individuals.

The generation time distribution is hard to estimate directly, given that it is often difficult to identify the exact timing of an infection event, let alone the timing of two sequential infection events required for inference of the generation time distribution. Generally, generation time distribution estimates are derived from serial interval (the time between symptom onset in a case and symptom onset of their infector) data, sometimes supplemented by partial data (e.g. time windows of exposure) on infection times in secondary cases. The serial interval and generation time distributions typically have similar means but different variances [9]. Additionally, serial intervals can be negative, unlike generation times [9]. Recent literature has suggested using forward-looking serial intervals (in which time is measured forwards from symptom onset in an infector) gives the same estimate of *R* as with the generation time distribution [10]. The optimal approach for inferring the generation time distribution from serial interval data will depend upon the joint relationship of the infectious distribution and the incubation period [11].

The observed serial interval and the generation time distribution can be affected by both censorship (given long serial intervals cannot be observed) [12] and by the epidemic dynamics at the time of measurement (given in an exponentially growing outbreak, there will be many more recent infecteds) [10].

In practice, in a novel outbreak, initial estimates of the generation time distribution are based on an analysis of the ‘first few hundred cases' [13–17], with little emphasis on characterizing heterogeneities among infected individuals. Such heterogeneity is the focus of this paper.

We derive a multi-type equivalent of the renewal equation which accounts for heterogeneity in transmission including variation in case isolation behaviour, symptomatic/asymptomatic infection and heterogeneity introduced owing to vaccine roll-out. We refer to the *R* derived through the multi-type approach that accounts for these sources of heterogeneity as the *multitype R*. We then explore how much the multi-type *R* differs from a *naive R* derived from a single-type branching process based on the generation time distribution of the *reference* group (the group of unvaccinated, non-isolating, symptomatic individuals, from which the generation time distribution is calculated).

We consider two applications: to Ebola virus disease (EVD) in Guinea in 2014–15, and to SARS-CoV-2 in the UK between March 2020 and January 2021, to illustrate the potential impact on *R* estimates of neglecting heterogeneities owing to case isolation and asymptomatic transmission. In the theoretical parts of the paper, we assume the growth rate is known, while in the application parts of the paper, we assume the growth rate can be estimated from incidence case time-series in real time.

## 2. Methods

### 2.1. Single-type renewal equation

For a single-type epidemic, the renewal equation gives the relationship between the expected incidence, or number of new infected individuals on time *t*,  E[I(t)], with the true number of incident cases a time *τ* ago, I(t−τ), the instantaneous reproduction number at time *t,* R(t) and the generation time distribution, as function of time *τ* since infection, ω(τ)*:* $E[I(t)]=R(t)\int_{0}^{\infty }\omega (\tau )I(t-\tau )d\tau \;$ [7]. The renewal equation assumes (i) deterministic growth of infection incidence, which will be locally exponential and (ii) that the generation time distribution remains fixed through calendar time.

With a constant growth rate *r* in the period $t-\tau_{\max}\;$ to *t*, the incidence I(t)  will grow exponentially^1^ $I(t)=ke^{rt}\;$. The relationship between the reproduction number and the growth rate *r* is then given by equation (2.1) [18–20]:

2.1 $$R=\;\frac{1}{\int_{0}^{\infty }\omega (\tau )e^{-r\tau }d\tau }.$$

This enables us to generate the relationship between the reproduction number and growth rate for various generation time distributions in a homogeneous epidemic. In this paper, we use the gamma distribution, because it is frequently used in fitting generation time distributions, and it is analytical tractabile. Assuming the generation time distribution is well described by a gamma distribution with shape *a* and rate *b*, the analytic equation for the reproduction number in terms of the epidemic growth rate is given by equation (2.2):

2.2 $$\omega (\tau )=\;\frac{b^{a}}{\Gamma (a)}\tau^{a-1}e^{-b\tau };\;\;R=\frac{{\left(r+b\right)}^{a}}{b^{a}}.$$

### 2.2. Multi-type renewal equation

Moving to a paradigm where there are *n* groups with generation time distribution for a case in group *i* given by $\omega_{i}(\tau )$, we consider the next generation matrix, an *n* × *n* matrix where $R_{\;j\to i}$ represents the average number of secondary cases in group *i* resulting from one index case in group *j*. The values of $R_{\;j\to i}$ in turn will depend on the overall susceptibility and infectiousness of each group, and the extent of assortativity between groups.

In this case, the renewal equation becomes multi-dimensional and takes the form given in equation (2.3). As above, this assumes that the growth rate *r,* the generation time distribution for each group and assortativity remain constant in the period up from time *t − τ*_max_ to time *t*:

2.3 $$\left(\begin{array}{c} I_{1}(t)\\ \vdots \\ I_{n}(t) \end{array}\right)=\int_{0}^{\infty }\left(\begin{array}{ccc} R_{1\to 1}\omega_{1}(\tau ) & \cdots & R_{n\to 1}\omega_{n}(\tau )\\ \vdots & \ddots & \vdots \\ R_{1\to n}\omega_{1}(\tau ) & \cdots & R_{n\to n}\omega_{n}(\tau ) \end{array}\right)\left(\begin{array}{c} I_{1}(t-\tau )\\ \vdots \\ I_{n}(t-\tau ) \end{array}\right)d\tau .$$

We can assume an exponential solution as for the single-type case, with a vectorized *k*, with elements *k_i_* corresponding to the steady-state proportion of infections occurring in group *i* (equation (2.4)):

2.4 $$\left(\begin{array}{c} I_{1}(t)\\ \vdots \\ I_{n}(t) \end{array}\right)=\left(\begin{array}{c} k_{1}\\ \begin{array}{c} \vdots \\ k_{n} \end{array} \end{array}\right)e^{rt}.$$

Substituting equations (2.4) into (2.3) yields an eigenvalue equation (equation (2.5)):

2.5 $$\left(\begin{array}{ccc} R_{1\to 1}\int_{0}^{\infty }\omega_{1}(\tau )e^{-r\tau }d\tau & \ldots & R_{n\to 1}\int_{0}^{\infty }\omega_{n}(\tau )e^{-r\tau }d\tau \\ \vdots & \ddots & \vdots \\ R_{1\to n}\int_{0}^{\infty }\omega_{1}(\tau )e^{-r\tau }d\tau & \ldots & R_{n\to n}\int_{0}^{\infty }\omega_{n}(\tau )e^{-r\tau }d\tau \end{array}\right)\left(\begin{array}{c} k_{1}\\ \begin{array}{c} \vdots \\ k_{n} \end{array} \end{array}\right)=\left(\begin{array}{c} k_{1}\\ \begin{array}{c} \vdots \\ k_{n} \end{array} \end{array}\right).$$

To infer the overall reproduction number *R*, we factorize the matrix into the product of the scalar reproduction number *R* and the normalized next generation matrix *M*, whose elements $M_{\;j\to i}$ give the relative risk posed to a member of group *i* by an infected member of group *j*. Rearranging gives equation (2.6), the multi-type equivalent to equation (2.1):

2.6 $$R=\frac{1}{\max\left\{\mathrm{eigen}\left(\begin{array}{ccc} M_{1\to 1}\int_{0}^{\infty }\omega_{1}(\tau )e^{-r\tau }d\tau & \ldots & M_{n\to 1}\int_{0}^{\infty }\omega_{n}(\tau )e^{-r\tau }d\tau \\ \vdots & \ddots & \vdots \\ M_{1\to n}\int_{0}^{\infty }\omega_{1}(\tau )e^{-r\tau }d\tau & \ldots & M_{n\to n}\int_{0}^{\infty }\omega_{n}(\tau )e^{-r\tau }d\tau \end{array}\right)\right\}}.$$

We assume that the matrix *M* can be simply expressed using: (i) the relative infectiousness of each group, *η_i_*, (ii) the relative susceptibility of each group, *ξ_i_*, and (iii) the assortativity between the groups, given by a matrix *A* whose elements *A_ij_* give the proportion of group *j*'s contacts which are made with individuals in group *i* (equation (2.7)). The relative infectiousness and susceptibility of each group are denoted relative to the most infectious and most susceptible group, respectively. We consider *η* to be determined by biological factors and fixed through the course of an outbreak:

2.7 $$M=\frac{\eta \xi^{T}⊙A}{\left|\eta \xi^{T}⊙A\right|}\;;\;\underset{i}{\sum }A_{ij}=1.$$

The deterministic formulation above means we only consider central *R* estimates. We address stochastic variation in the application part of our work.

### 2.3. Mathematical treatment of heterogeneity

In this paper, we use the same formalism as outlined by Fraser in [7], where transmissibility β(t,τ) measures the instantaneous rate of onward infections generated by a primary case as a function of time since their infection τ and calendar time *t*. As in [7], we assume β(t,τ) can be expressed as β(t,τ)=R(t)ω(τ) where R(t) is the instantaneous reproduction number which depends only on calendar time, and ω(τ) is the generation time distribution which depends only on time since infection. Transmissibility will reflect both the pathogen shedding rate and the extent of contacts an infected person has over the course of their infection.

We consider three scenarios relevant to many infectious diseases, but in particular to SARS-Cov-2: heterogeneity owing to (i) the isolation of symptomatic cases on symptom onset; (ii) the presence of asymptomatic carriers; and (iii) differential transmission potential of vaccinated individuals, which will be increasingly important as vaccination is rolled out. In all scenarios, we consider epidemic growth rates of −0.3, −0.15, 0, 0.15 and 0.3 d^−1^ which correspond to halving times of 2.3 and 4.6 days, steady-state and doubling times of 4.6 and 2.3 days, respectively. We consider reference group sizes corresponding to 20%, 50% and 80% of the total population. In (i) and (ii), we assume homogeneous mixing between groups, while in (iii) we allow for assortativity in mixing. In all scenarios, we assume the incidence of infection is known accurately, with no reporting delays, which would enable calculation of the epidemic growth rate. We also assume the generation time distribution is well characterized for the reference group. For details on how the generation time distribution of the non-reference group is constructed in each case, see the electronic supplementary material, methods.

We parameterize the assortativity matrix A in a similar way to [21], which described HIV transmission by considering mixing within and between sexual activity groups via the contact rates of members from each group *c*_1_ and *c*_2_; the proportion of the population in each group, *p_1_* and *p_2_*_,_ and an assortativity parameter *δ*. We do not explore the effect of heterogeneity in contact rate by individuals in each group, making the simplifying assumption that contact rates are uniform independent of group, resulting in the parameterization given in equation (2.8) where *p*_1_ is the smaller population such that all matrix elements are less than or equal to 1:

2.8 $$A(\delta )=\left(\begin{array}{cc} \delta & (1-\delta )\frac{\;p_{1}}{\;p_{2}}\\ 1-\delta & 1-(1-\delta )\frac{\;p_{1}}{\;p_{2}} \end{array}\right).$$

With homogeneous mixing, all matrix elements in a row will be the same, as the extent of interaction any individual has with group *i* is determined solely by the proportion of the population that is in group *i.* For plotting, we vary *δ* in a two-part linear manner, from 0 (disassortative) to *p*_1_ (homogeneous) from the left of the *x*-axis to the middle, and from *p*_1_ to 1 (assortative) from the middle of the *x*-axis to the right-hand side. This standardizes homogeneous mixing at the centre of the *x*-axis.

### 2.4. Equivalent single-type formulation

The single-type formalism of the reproduction number provides a more straightforward means of inferring the reproduction number. Additionally, existing software packages used for epidemic analysis will typically only work with single-type renewal processes, so there is a benefit to expressing the multi-type renewal processes as an equivalent single-type.

Equations (2.3) and (2.4) can be re-written as equation (2.9):

2.9 $$\left(\begin{array}{c} I_{1}(t)\\ \vdots \\ I_{n}(t) \end{array}\right)=\;R\int_{0}^{\infty }\left(\begin{array}{ccc} M_{1\to 1}\omega_{1}(\tau ) & \cdots & M_{n\to 1}\omega_{n}(\tau )\\ \vdots & \ddots & \vdots \\ M_{1\to n}\omega_{1}(\tau ) & \cdots & M_{n\to n}\omega_{n}(\tau ) \end{array}\right)\left(\begin{array}{c} k_{1}\\ \vdots \\ k_{n} \end{array}\right)e^{r(t-\tau )}d\tau .$$

The total number of newly infected individuals is then

2.10 $$\begin{array}{rl} I(t)=\; & \underset{i}{\sum }R\int_{0}^{\infty }\underset{j}{\sum }k_{j}M_{\;j\to i}\omega_{j}(\tau )e^{r(t-\tau )}d\tau \\ = & R\int_{0}^{\infty }\tilde{\omega }(\tau )e^{r(t-\tau )}d\tau \;a, \end{array}$$

where $\tilde{\omega }(\tau )$ is given by equation (2.11), in which *C* is chosen as a normalizing constant such that $\int_{0}^{\infty }\tilde{\omega }(\tau )d\tau =1$:

2.11 $$\tilde{\omega }(\tau )=C\underset{i,j}{\sum }k_{j}M_{\;j\to i}\omega_{j}(\tau )=C\underset{j}{\sum }\left(k_{j}\omega_{j}(\tau )\underset{i}{\sum }M_{\;j\to i}\right).$$

Equation (2.10) shows that the multi-type renewal equation can be written as single-type renewal equation with a weighted mean generation time distribution. The weighting is given by the overall relative reproduction number of group *j*, and the *j*^th^ element of the eigenvector, which corresponds to the equilibrium proportion of infections that occur in group *j*. It is worth noting that in practice during an epidemic, the true equilibrium proportion of infections occurring in each group may not be known exactly. This does not affect our theoretical results as we derive the equilibrium proportion of infections directly from the eigenvector of the mixing matrix shown in equation (2.9), and we assume no importations or stochasticity. Given this, the weighted single-type approach derived in equation (2.10) will return the same multi-type *R* as derived in equation (2.6).

### 2.5. Use in EpiEstim for application to COVID-19 in the UK and Ebola virus disease in Guinea

Equations (2.1) and (2.6) describe the relationship between the instantaneous reproduction number, the growth rate and the generation time distribution of different groups. In practice, the growth rate is not directly observed, but can be estimated from the incidence time-series. This leads to uncertainty in the growth rate estimates, and in turn the corresponding reproduction number estimates, which are not represented in the equations above.

The R package EpiEstim implements estimation of the instantaneous reproduction number, based on an incidence time-series and a discrete generation time distribution. We used EpiEstim given its frequent use in real-time epidemic modelling, and it being the best-performing package according to a recent review of methods to estimate *R* in real time by Gostic *et al.* [8]. EpiEstim uses a single-type renewal equation to estimate the posterior distribution of the instantaneous reproduction number, capturing uncertainty in the estimates. We therefore use EpiEstim to compare the naive *R* estimates, with the multi-type *R* based on appropriately weighted single-type generation time distribution (equivalent to the multi-type approach). EpiEstim makes several important underlying assumptions including that: (i) a constant proportion of all infections are detected; (ii) aside from cases on the first day, no cases are imported (such that each case could be attributed to a previous case in the time-series); (iii) that the generation time is constant throughout the outbreak; and (iv) that the offspring distribution is Poisson distributed.

In all applications, *R* values were estimated over sliding weekly windows, over which we assume *r* is constant. This is likely to be a reasonable approximation unless there are very rapid changes in policy and short generation times. We assume that the generation time distribution is known only for the unvaccinated, symptomatic and non-isolating reference group. Moreover, we assume that the proportion of individuals in each group is constant.

We estimate the instantaneous reproduction number for EVD case data from Guinea between March 2014 and July 2016, with data taken from [22]. We use a generation time with mean of 15.3 and standard deviation of 9.3 days following [23]. This is assumed to be reflective of a non-isolating cohort. We assume that isolation occurs at the point of hospitalization at 14.9 days after infection (56.5% of the way into a non-isolated transmissibility profile), based on the sum of the mean incubation period and the mean delay from symptoms to hospitalization given in [24]. While the delay to hospitalization follows a distribution, here we use the mean as a single time-point delay to illustrate the impact of accounting for isolation in a simple way. We assume that 34% of infected individuals are hospitalized (J.T. Unwin, A. Cori, N. Imai, K.A.M. Gaythorpe, S. Bhatia, L. Cattarino, C.A. Donnelly, N.M. Ferguson, M. Baguelin 2022, unpublished data), and that individuals who seek and do not seek hospitalization mix homogeneously.

We estimate the instantaneous reproduction number of SARS-CoV-2 using time-series of COVID-19 deaths in the UK from March 2020 to January 2021. We use incidence of deaths rather than cases because ascertainment of deaths varies less through time, especially given in the early months of the epidemic testing capacity was being built up. Given *R* estimates from the renewal equation are robust to constant under-reporting, we assume that reported deaths remained a constant fraction of cases over time. The rationale mimics that used by Nouvellet *et al.* in [25]. Note that owing to a delay between infection and death, the resulting estimates of *R* will be lagged and smoothed—the average delay from infection to symptom onset is estimated to be around 5.5 days in the UK [26], while estimates of the delay from symptom onset to death range from around 13 days [27] to 18 days [28]. Other papers have demonstrated inference of *R* accounting for lagged metrics, for example [29]. Daily UK COVID-19 deaths were taken from the government's coronavirus data repository [30]. We assumed a generation time in the absence of isolation with mean of 5.29 days and standard deviation of 2.08 days as in [31], and that 63% of individuals are symptomatic. In a sensitivity analysis (see the electronic supplementary material, methods), we further account for uncertainty in the mean and standard deviation of the generation time. Note that in what follows we describe scenarios in which the poorly characterized group have a lower reproduction number or a longer generation time distribution as ‘optimistic’ scenarios and vice versa. As such, the use of terms ‘optimistic’ and ‘pessimistic’ pertain to the transmissibility profile of the non-reference group and *not* the impact on inferred *R*.

## 3. Results

### 3.1. Symptomatic isolation and non-isolation

**Figure 1.**
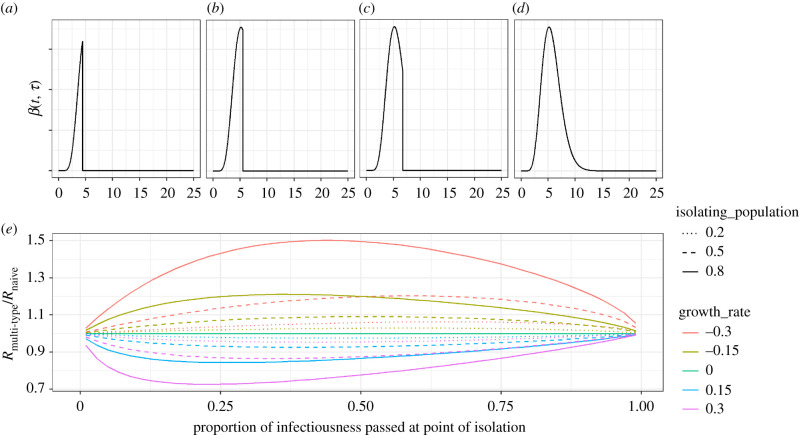
(*a–c*) Transmissibility profiles for individuals isolating 25%, 50% and 75% through their infection, respectively. (*d*) Transmissibility profile for non-isolating individuals. (*e*) The relative difference in *R* inferred by considering a multi-type branching process as compared to a naive single-type branching process, accounting for isolation of a subset of cases by: (i) the amount of infectiousness that has passed at the time of isolation (*x*-axis), (ii) the size of the isolating population (linetype), and (iii) the growth rate of the epidemic (colour). All graphs correspond to a reference generation time distribution which is gamma distributed with a mean of 5.29 days and a standard deviation of 2.08 days.

Isolation of symptomatic cases on onset will necessarily reduce the transmissibility profile for those who isolate (for example, figure 1*a–c—*corresponding to isolation 25%, 50% and 75% into the generation time distribution, compared to figure 1*d*—corresponding to no isolation). In a growing epidemic, isolation will mean the multi-type *R* is lower than the naive *R* (using the single-type approach) and vice-versa for shrinking epidemics (figure 1*e*). This error is higher with higher growth/reduction rates, as well as with higher isolating populations (figure 1*e*). The error is zero at the extremes: if isolation occurs immediately upon infection; or if isolation occurs following all infection (figure 1*e*). This is because if isolation occurs immediately, isolators do not contribute to the infectious pool, so the weighting of the non-isolating group in equation (2.11) is zero. Likewise, if isolation occurs following the infectivity period, it is equivalent to no isolation occurring.

Overall, over the parameter space explored, the multi-type *R* is a maximum of 1.5 times higher than the naive *R* when the epidemic growth rate is −0.3 d^−1^, and isolation occurs among 80% of the population 43% of the way into the transmissibility profile. The multi-type *R* is 0.73 times lower than the naive *R* when the epidemic growth rate is 0.3 d^−1^, and isolation occurs among 80% of the population 23% of the way into the transmissibility profile (figure 1*e*).

**Figure 2.**
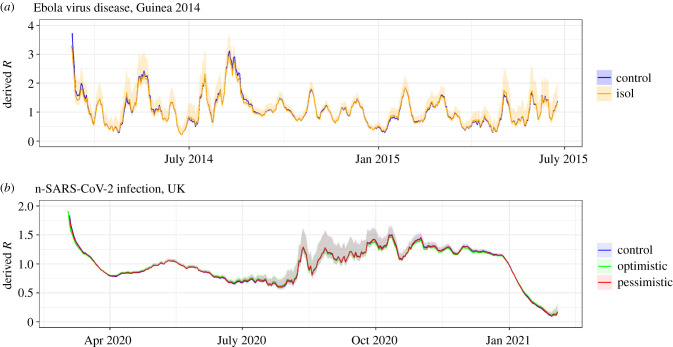
(*a*) Time-varying *R* values for the EVD outbreak from March 2014 to July 2015 in Guinea from EpiEstim, derived using a naive generation time distribution (blue), and a weighted generation time distribution (equivalent to a multi-type branching process) accounting for case isolation (orange). Credible intervals are shown for the weighted generation time distribution, demonstrating that consideration of case isolation makes little difference to the overall estimated *R*. The reference generation time distribution is assumed to be gamma distribution with a mean of 15.3 days and a standard deviation of 9.3 days. We assume 36% of cases isolate with isolation occurring 55% of the way into the infectious distribution. (*b*) Time-varying *R* values for the SARS-CoV-2 outbreak from March 2020 to February 2021 in the UK, based on optimistic and pessimistic assumptions around isolation. In both cases, we assume 63% of infections are symptomatic and the reference generation time distribution (corresponding to non-isolating symptomatic individuals) is gamma distributed with a mean of 5.29 days and a standard deviation of 2.08 days. Optimistic assumptions are that isolation occurs among 75% of symptomatic infection after 30% of infectivity has passed. Pessimistic assumptions are that isolation occurs in 25% of symptomatic infection after 70% of infectivity has passed. In both cases, *R* estimates are based on sliding weekly windows.

Case isolation heterogeneity was considered in the context of the EVD outbreak in Guinea between March 2014 and July 2015 in figure 2*a*, and for SARS-CoV-2 between March 2020 and February 2021 in figure 2*b*. Both applications confirm that case isolation has limited impact on the overall derived *R*.

The reason for the limited impact can be seen by considering the equivalent single-type renewal process airing from equations (2.10) and (2.11). The isolating group produces fewer onward infections than the non-isolating group (manifesting in the sum over *M_j_*_→*i*_) which means their contribution to the weighted generation time distribution is lower. The impact of isolation is particularly low in the case of a growing epidemic. This is because the generation time distribution undergoes exponential discounting (equation (2.10)) which reduces the relative contribution of late transmission in a growing epidemic—the part of the generation time distribution that case isolation has an impact on.

### 3.2. Symptomatic and asymptomatic transmission

We compared the naive *R* estimates from the single-type model (equation (2.1)) using the generation time distribution of symptomatic individuals with those from the multi-type model assuming asymptomatics have a different generation time distribution (equation (2.6)). We explore this difference as we vary the relative reproduction number of symptomatic and asymptomatic individuals.

**Figure 3.**
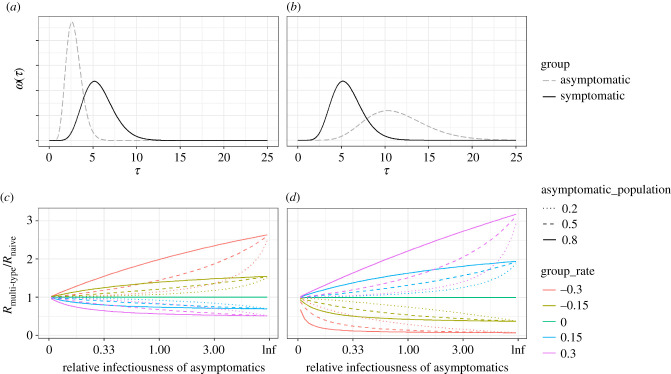
(*a,b*) Explored generation time distributions for symptomatic and asymptomatic individuals. In (*a*), the generation time distribution of asymptomatics is half that of symptomatics, whereas in (*b*), the generation time distribution of asymptomatics is double that of symptomatics. In both cases, the symptomatic (reference) generation time distribution has a mean of 5.29 days and a standard deviation of 2.08 days. (*c,d*) The relative difference in *R* obtained using a multi-type branching versus a naive single-type branching process, accounting for asymptomatic transmission by: (i) the relative infectiousness of asymptomatics (*x*-axis), (ii) the size of the asymptomatic population (linetype), and (iii) the growth rate of the epidemic (colour); (*c*) corresponds to the generation time distributions given in (*a*) while (*d*) corresponds to those given in (*b*).

If the generation time distribution of asymptomatic carriers is longer than that of symptomatic carriers (figure 3, right), the multi-type *R* will exceed the naive *R* in a growing epidemic and will be lower than the naive *R* in a declining epidemic. This trend is reversed for dynamics in which the generation time distribution of asymptomatic carriers is shorter than that of symptomatic carriers (figure 3, left). The error in inferred *R* becomes greater at higher absolute values of growth rate, with higher asymptomatic infection rates, and with higher relative reproduction numbers of asymptomatic individuals (figure 3*c,d*).

With the extent of variation explored, the multi-type *R* exceeded the naive *R* by up to three times when the generation time distribution of asymptomatics was twice as long as that of symptomatics, and asymptomatics were responsible for all onward infection (figure 3*d*). While this represents a relatively extreme scenario, it may be relevant for pathogens with early onset of symptoms among symptomatic cases but late onset of infectiousness, by which point symptomatic individuals may have reduced their contacts substantially, meaning asymptomatic individuals would be more responsible for onward transmission.

**Figure 4.**
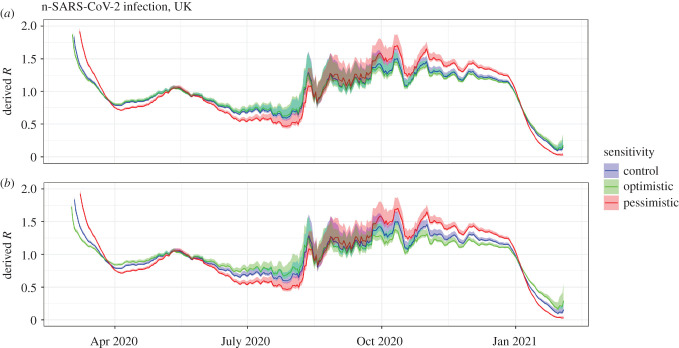
Derived value of *R* based on UK deaths using a multi-type and a naïve branching process for (*a*) asymptomatic transmission, and (*b*) asymptomatic transmission and symptomatic isolation together. We assume homogeneous mixing for both cases. Optimistic assumptions correspond to asymptomatics with half the generation time distribution of symptomatics and half the reproduction number. Pessimistic assumptions correspond to asymptomatics having twice the generation time distribution of symptomatics and twice the reproduction number. For case isolation which is included in (*b*), the optimistic and pessimistic assumptions given in figure 2 apply. Optimistic assumptions are that isolation occurs among 75% of symptomatic infection after 30% of infectiousness has passed. Pessimistic assumptions are that isolation occurs in 25% of symptomatic infection after 70% of infectiousness has passed. We assume 63% of infections are symptomatic and the reference generation time distribution (corresponding to non-isolating symptomatic individuals) is gamma distributed with a mean of 5.29 days and a standard deviation of 2.08 days. In both cases, *R* estimates are based on sliding weekly windows.

Potential asymptomatic transmission of SARS-CoV-2 in the UK is considered in figure 4*a*. A different asymptomatic generation time distribution can result in a substantial difference in the inferred *R*. We explore an optimistic case, in which asymptomatic transmitters have half the reproduction number and have half the generation time distribution as symptomatic counterparts, and a pessimistic case, in which asymptomatic transmitters have a prolonged (twice) generation time distribution and twice the reproduction number of their symptomatic counterparts.

In figure 4*b*, we consider a three-type branching process consisting of asymptomatic carriers, symptomatic carriers who isolate and symptomatic carriers who do not isolate. Results were broadly similar when accounting for uncertainty in the mean and standard deviation of the generation time distribution (see the electronic supplementary material), though as expected uncertainty in the *R* estimates was greater.

### 3.3. Vaccinated and unvaccinated groups with assortative and disassortative mixing

In what follows, we explore scenarios in which vaccination reduces susceptibility to infection and results in a simultaneous and equal reduction in both the generation time distribution and the peak transmissibility among the subset of vaccinated individuals who still get infected. In previous examples, we have considered homogeneous mixing between groups. However, assortativity of mixing may be relevant to vaccination, given vaccination policy may target age cohorts [32,33] or because of differential vaccine uptake [34,35].

**Figure 5.**
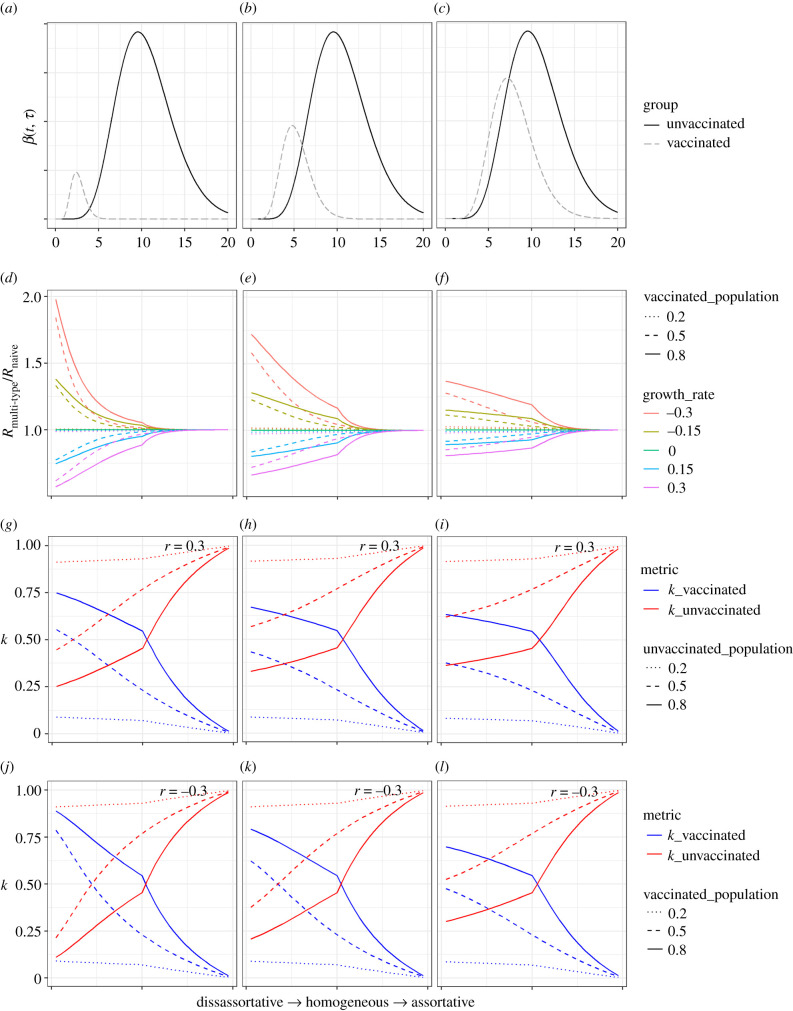
(*a–c*) Explored scenarios for vaccine impact on the transmissibility profile; (*d–f*) the relative difference in *R* obtained using a multi-type branching proves versus a naive single-type branching process with unvaccinated reference group, accounting for vaccinated individuals by (i) the extent of assortativity (*x*-axis), (ii) the size of the asymptomatic group (linetype), and (iii) the growth rate of the epidemic (colour). We assume that vaccination reduces susceptibility by 70% in all explored scenarios; (*g–i*) elements of the eigenvector corresponding to the vaccinated and unvaccinated groups in a growing epidemic with *r* = 0.3, normalized such that their sum is 1; (*j–l*) elements of the eigenvector corresponding to the vaccinated and unvaccinated groups in a declining epidemic with *r* = −0.3, normalized such that their sum is 1. For graphs (*d*–*l*), the *x*-axis is a two-part discontinuous linear scale from disassortative to homogeneous and from homogeneous to assortative.

We assume vaccination reduces individuals' susceptibility to infection by 70%. We trial three different simultaneous decreases in the duration and peak height of transmissibility of vaccinated individuals: by 25%, 50% and 75%, corresponding to reductions in individual reproduction number of 44%, 75% and 93.75% relative to the unvaccinated group (figure 5*a–c*). The difference between the naive and multi-type *R* is highest for disassortative mixing, given with disassortative mixing a substantial share of transmission passes through the vaccinated group, whose generation time distribution is not included in the unweighted single-type approach. The difference in inferred *R* reduces to zero in the limit of totally assortative mixing, as this represents two isolated outbreaks, for which the epidemic growth rate is totally driven by the unvaccinated group (figure 5*d*–*f*). The difference in inferred *R* reduces especially quickly as the contribution of the unvaccinated group (for whom the generation time distribution is well characterized) increases. This can be seen by considering the relative sizes of the elements of the eigenvector in equation (2.5) corresponding to the proportion of infections that are in the vaccinated and unvaccinated groups (shown in figure 5*g*–*i* for a growing epidemic, and in figure 5*j*–*l* for a shrinking epidemic). The greater the proportion of infections (or *k*-value) in the unvaccinated group, the closer the multi-type *R* is to the naive *R*.

## 4. Discussion

In this paper, we have shown how and when heterogeneity in the generation time distribution can distort estimates of the reproduction number. While in the parameter range we explored, the impact on the inferred reproduction number is limited in the case of symptomatic case isolation, it can be considerable if asymptomatic or vaccinated individuals have particularly different generation time distributions from unvaccinated, symptomatic individuals (for whom the generation time distribution will be best characterized). The difference in inferred *R* will be smaller for lower growth rates; where the poorly characterized groups represent a small part of the population; or where there is highly assortative mixing between groups.

There are several assumptions underlying this work. In the theoretical part, we assume a known, fixed growth rate, and that there is no heterogeneity in transmissibility within groups, only between. In our applications, we assumed generation times and assortativity were fixed over the course of the outbreak, and that a constant proportion of individuals were symptomatic or isolators. In reality, generation time distributions change over the course of an outbreak, e.g. because of increasing competition between infectors [36], the implementation of interventions, or changes in behaviours including increased adherence to symptomatic isolation [37].

In using the renewal equation, we assumed transmissibility could be separated into a reproduction number, depending only on calendar time *t*, and a generation time distribution depending only on time since infection, *τ*. However, behaviour is likely to change as an epidemic progresses, for instance through a reduction in out-of-household contacts, which may cause the generation time distribution to change independently of case isolation.

For the multi-group case, we assumed that relative infectiousness and susceptibility between groups remained constant through time. This too is a simplifying assumption: interventions such as the adoption of face coverings may alter the relative susceptibility and infectiousness of groups, especially if there is a correlation between the group and the adoption of certain behaviours. For example, isolation on symptom onset may well be correlated with compliance to mask-wearing and handwashing.

Various papers have discussed the use of *R* in public health policy. In the last 18 months, *R* has been central to understanding the state of the epidemic in the UK [38] and was one of the key metrics assessed in recommendations from the Scientific Pandemic Influenza Group on Modelling consortium [39]. While our study shows that disregarding heterogeneity in transmission does not affect the central question of whether *R* is greater than or less than 1 in an epidemic, we find that the further *R* is from 1 the greater the potential error when ignoring such heterogeneity. This could have multiple consequences, including: (i) underestimating the likely attack rate and therefore the epidemic burden [40]; (ii) inadequate planning of vaccination to achieve herd immunity [41,42]; (iii) misinformed decisions in favour of or against a given control measure which is effective at certain levels of *R* but not others [43,44]; and (iv) wrongly estimating the end date of the epidemic, which has potential logistical implications on international assistance or aid [45].

Multiple pathogens have been demonstrated to have both symptomatic and asymptomatic clinical courses, including important epidemic viruses SARS-CoV-2 [46], Influenza [47], EVD [48] and Middle East respiratory syndrome (MERS) [49]. Additionally, previous work has shown that a difference in the generation time distribution of asymptomatic versus symptomatic carriers of COVID can lead to biased estimates of the effective reproduction number [50].

The transmissibility profile of an individual depends principally on the extent and duration of viral shedding, and their effective contact rate. Symptom presentation may impact both variables. Viral shedding will itself depend on individual viral load, and the efficiency and duration of viral expulsion. Viral load studies for influenza infection have shown that asymptomatic and paucisymptomatic cases had 1-2 log_10_ fewer copies of viral RNA than symptomatic cases and shorter shedding times [51]. Similarly, studies on MERS found the duration of polymerase chain reaction-positivity increased with disease severity [52]. Symptoms themselves also increase viral expulsion: a cough can produce an estimated 3000 droplets and a sneeze an estimated 40 000 [53]; both far more efficient shedding processes than breathing or talking [54].

There have been varying conclusions from studies on the difference in viral load between symptomatic and asymptomatic infections in SARS-CoV-2 infection. Where some studies have found viral load to be similar between symptomatic and asymptomatic SARS-CoV-2 patients [55,56], others have found statistically significant differences in viral load [57,58] and clearance time [59,60], or that shedding duration increases with disease severity [61]. A further study in Catalonia has found severity to be positively correlated with viral load, and that higher viral loads led to a greater extent of onward transmission [62]. A recent literature review including 79 studies on SARS-CoV-2 concluded that the sum of evidence suggests viral load is similar between symptomatic and asymptomatic individuals, most studies ‘demonstrate faster viral clearance among asymptomatic than those who are symptomatic’ [63, p. e19]. A further systematic review of the reproduction number and secondary attack rate suggested asymptomatic cases were around one-seventh as infectious as symptomatic individuals [64]. Conversely, symptomatic SARS-CoV-2 infecteds are likely to reduce their contacts following onset: in the UK, isolation of 10 days is mandated for individuals developing symptoms (and subsequently receiving a positive test for) of COVID-19, and for their households [65].

Early studies demonstrate vaccinated individuals infected with SARS-CoV-2 have a lower viral load than unvaccinated individuals [66,67] and lower susceptibility to SARS-CoV-2 infection [68]. Reduction in peak viral load and viral clearance times have also been demonstrated with oral and inactivated poliovirus vaccine [69,70]. Understanding the generation time distribution of multiple groups becomes increasingly important with disassortative mixing, for instance when estimating the reproduction number of sexually transmitted infections in heterosexual contact networks with human papillomavirus, for which vaccination uptake was previously limited to females.

As vaccines against SARS-CoV-2 continue to be rolled out over the coming months, understanding the impact of the vaccine on susceptibility and transmissibility will be increasingly important for accurate inference of *R*. Given the vaccine schedule is broadly age-prioritized, mixing by vaccination status will be more assortative.

Estimating the contemporaneous generation time distribution should be regarded as similarly important to estimation of the reproduction number itself, which currently occupies the work of academic modelling groups worldwide for SARS-CoV-2. Better capturing the heterogeneities of the generation time distribution will become increasingly important as vaccination is rolled out, as well as with the emergence of new strains which may exhibit different transmissibility profiles. Upcoming SARS-CoV-2 challenge trials in the UK should enable detailed analysis of viral load profiles and symptomatic rates, which can inform updated generation time distributions [65].

While estimation of the generation time distribution is necessarily a time-consuming endeavour, testing systems should integrate additional epidemiological information in tandem with their test and trace protocols. Updated estimates of the serial interval could be obtained by requiring test applicants to supply their symptom onset date, with linkage to traced contacts should they also enter the testing system. For a more direct means to estimate changes in the generation time distribution, or indeed the incubation period, individuals could be asked for dates of contact with known infected in the previous week, and this too linked with contacts who enter the test and trace system.

## Data Availability

Data on Ebola cases are taken from: Heterogeneities in the case fatality ratio in the West African Ebola outbreak 2013–2016 [22]. Data on SARS-CoV-2 deaths in the UK taken from the Government dashboard: https://coronavirus.data.gov.uk/. Both data and code are available in the Github repository: https://github.com/willgreen236/Heterogeneity_transmission.git.
